# Second-line failure and first experience with third-line antiretroviral therapy in Mumbai, India

**DOI:** 10.3402/gha.v7.24861

**Published:** 2014-07-30

**Authors:** Samsuddin Khan, Mrinalini Das, Aristomo Andries, Alaka Deshpande, Homa Mansoor, Peter Saranchuk, Petros Isaakidis

**Affiliations:** 1 Médecins Sans Frontières, Mumbai, India; 2 Infectious Diseases Department, Mahatma Gandhi Medical College, Mumbai, India; 3 Southern Africa Medical Unit (SAMU), Médecins Sans Frontières, Cape Town, South Africa

**Keywords:** viral load, HIV, adherence, counselling, antiretroviral therapy, genotyping, India

## Abstract

**Background:**

There are limited data on the failure of second-line antiretroviral therapy (ART) and the use of third-line ART in people living with HIV in resource-limited settings. Since 2011, the Médecins Sans Frontières (MSF) HIV/tuberculosis programme in Mumbai, India, has been providing third-line ART to patients in care.

**Objective:**

To describe the experiences and programmatic challenges during management of suspected second-line ART failure and third-line ART therapy for patients living with HIV, including the use of HIV viral load (VL) testing.

**Design:**

This was a retrospective, observational cohort study of patients with suspected second-line ART treatment failure, who were followed for at least 12 months between January 2011 and March 2014.

**Results:**

A total of 47 patients with suspected second-line failure met the inclusion criteria during the study period. Twenty-nine of them (62%) responded to enhanced adherence support, had a subsequent undetectable VL after a median duration of 3 months and remained on second-line ART. The other 18 patients had to be initiated on a third-line ART regimen, which consisted of darunavir–ritonavir, raltegravir, and one or more appropriate nucleoside or nucleotide reverse transcriptase inhibitors, based on the results of HIV genotype testing. Of the 13 patients for whom follow-up VL results were available, 11 achieved virological suppression after a median duration of 3 months on third-line ART (interquartile range: 2.5–3.0). No serious treatment-related adverse events were recorded.

**Conclusions:**

With intensive counselling and adherence support in those suspected of failing second-line ART, unnecessary switching to more expensive third-line ART can be averted in the majority of cases. However, there is an increasing need for access to third-line ART medications such as darunavir and raltegravir, for which national ART programmes should be prepared. The cost of such medications and inadequate access to VL monitoring and HIV genotype testing are currently major barriers to optimal management of patients failing second-line ART.

India is home to approximately 2.1 million people living with HIV (1). Of the 0.6 million people living with HIV and on antiretroviral therapy (ART) in 2012, approximately 5,503 (0.9%) were receiving second-line ART and none were on third-line ART under the National AIDS Control Programme.

Because a certain percentage of patients living with HIV fail their ART regimens every year, and there is the possibility of transmission of drug-resistant HIV (2, 3), a gradual rise in the number of patients failing second-line ART regimens can be expected, as well as a growing need for access to third-line ART in the future. Furthermore, the cost of a third-line regimen represents a major challenge, because it is nearly 15 times higher than that of a first-line ART regimen and over six times that of a typical second-line regimen (4).

Routine HIV viral load (VL) testing is an indispensable requirement in any ART programme. The World Health Organization (WHO) recommends VL monitoring as the preferred approach to provide an early and more accurate indication of treatment failure, thereby reducing the accumulation of drug-resistance mutations and improving clinical outcomes (5). However, VL testing is not routinely offered in many countries owing to its cost and lack of availability.

To date, there are limited data describing experiences of routine VL monitoring in the contexts of patients living with HIV who have suspected second-line ART failure and the use of third-line ART in resource-limited settings. To address this knowledge gap, this study describes the experiences and programmatic challenges during treatment and VL testing of patients living with HIV who are suspected of second-line ART failure and those needing third-line ART in Mumbai within a non-governmental organization (NGO)-run programme that provides ART and VL testing free of charge.

## Methods

### Study design

This was a retrospective, observational cohort study of patients living with HIV who had suspected failure of second-line ART using data collected under routine programmatic conditions.

### Setting and study population

Since 2006, Médecins Sans Frontières (MSF) has been operating an HIV/tuberculosis (TB) programme in Mumbai, India (6), for those unable to access essential HIV care and treatment services elsewhere. During the study period, the size of the entire clinic cohort was approximately 290 patients. One of the main objectives of the MSF programme has been to provide free second-line or third-line ART as appropriate to patients living with HIV who are not able to access these through national ART centres. These patients living with HIV are referred from government ART centres, public–private ART centres, and a network of community NGOs. The programme initially started providing third-line ART in 2011. Patients were eligible for inclusion in this study if they were suspected of failing a second-line ART regimen that included a protease inhibitor (PI) and 2–3 nucleoside or nucleotide reverse transcriptase inhibitors (NRTIs), and they were followed for at least 12 months between January 2011 and March 2014.

### Definitions and treatment protocol

Second-line ART was defined as the regimen used for treatment of patients living with HIV who failed a first-line regimen, and typically it would consist of a PI (e.g. atazanavir or ritonavir) and two or three NRTIs (e.g. lamivudine and tenofovir±zidovudine). Third-line ART regimen in the MSF programme included the second-generation PI darunavir boosted with ritonavir (DRV/r) and the integrase inhibitor raltegravir (RAL), together with one or more NRTIs likely to be effective on the basis of HIV genotyping results. As part of routine clinical care, HIV VL and CD4 cell count testing was carried out for each patient every 6 months in the MSF programme. An undetectable VL was defined as a result with fewer than 50 copies/ml. Treatment failure was defined as a VL result >5,000 copies/ml in two consecutive results in a 3-month time frame, as previously recommended by WHO (7).

Patients in the programme were suspected of failing second-line ART if they had received and failed a first-line regimen, were subsequently treated with a second-line regimen for at least three consecutive months, and then had a single VL test result measuring >5,000 copies/ml. All patients suspected of failing second-line ART underwent a thorough assessment, including structured client-centred adherence counselling and repeated VL measurement after 3 months of good adherence. The assessment involved a multidisciplinary team consisting of an HIV physician, psychologist, social worker, and nurse. Each member of the team had special predefined tasks and responsibilities (Box 1). The time to virological suppression, if achieved, was measured and reported.

Box 1 **Predefined tasks and responsibilities of members of the multidisciplinary team assessing those suspected of failing second-line ART.**

***HIV Physician:*** Physicians perform a detailed history and examination at the first visit, and they arrange for a number of laboratory tests, including complete blood count, alanine transaminase, serum creatinine, serum bilirubin, blood sugar, serum lipids, Western blot (for HIV-1 and 2), HIV-1 viral load (VL), CD4 count, chest X-ray, abdominal ultrasound, and fundoscopy, as well as a pregnancy test in females. Second-line ART is restarted only after ruling out opportunistic infections. Any treatment-related adverse events are symptomatically managed. Initially, the patient is assessed every two weeks for the first three visits and then monthly. At every visit, adherence is reinforced, and after 2–3 months of good adherence, VL testing is repeated. VL and CD4 count testing are repeated every 6 months. If VL suppression is not achieved, genotyping is performed, and a treatment regimen is designed based on the genotyping results.

***Psychologist:*** Every patient is evaluated at baseline, including assessment with the PHQ9 Patient Depression questionnaire. Baseline HIV knowledge is tested using 30 basic HIV questions: a score of <10 is defined as poor knowledge, 11–20 as average knowledge, and 21 or more as good knowledge. Barriers for adherence are identified. The patients see the psychologist every two weeks for the first three visits, and then every month.

***Social Worker:*** Every patient receives a social assessment at their first visit, during which social barriers for adherence are identified and solutions discussed with the patient.

***Nurses:*** Dosage and timing of all pills are explained to all patients. A pill count is done at every visit, and the percentage of pills taken is entered into a file. Pillboxes are used for patients who have difficulties with timing of their ARVs.

The adherence levels of all patients were assessed with patient self-reports. These levels and any adherence concerns were recorded by the team following individual interviews. In patients observed to have adherence issues, the team provided education to the patient about HIV, VL and CD4 testing, and treatment failure. Psychological and emotional support were offered to the patients during treatment, and they were also referred to other NGOs and services as needed.

A two-log decrease in the repeat VL measurement 3 months after interventions to support adherence in those suspected of failing a second-line regimen was defined as virological suppression, and these patients were continued on second-line ART.

Those patients who failed to achieve virological suppression were investigated for antiretroviral (ARV) resistance using genotyping with the ViroSeq HIV-1 genotyping system method (Celera Diagnostics, Alameda, CA, USA); the instrument used was the ABI 310 Genetic Analyzer (Applied Biosystems, Carlsbad, CA, USA). The Stanford genotypic resistance interpretation algorithm was used to analyse resistance of HIV in these patients. Patients in need of a third-line regimen were prescribed the regimen based on genotyping results.

### Data collection and analysis

Demographic and clinical information of all patients living with HIV was recorded in patient files. The clinical data that were routinely collected for each patient, including treatment and laboratory data, were entered into an electronic database. A full-time data manager routinely supervised data entry for accuracy and completeness. Data from all patients suspected of second-line failure between January 2011 and March 2014 were included in the analyses. Descriptive statistics were used to analyse the data of these patients using SPSS (Release 20, 2011; SPSS, Inc., Chicago, IL, USA).

### Ethics

The study satisfied the criteria for reports using routinely collected programmatic data set by the MSF independent Ethics Review Board in Geneva, Switzerland. As this was a study of routinely collected monitoring data, informed consent from the patients was not obtained. The named ethics committee specifically approved the study and waived the need for consent.

## Results

### Patient characteristics

A total of 47 patients living with HIV were suspected to have failed second-line ART during the study period. The median age of patients was 40 years (interquartile range (IQR): 35–46), and almost three-fourths of them (35/47) were male. The median baseline CD4 count for these patients at the time of enrolment was 110 cells/µl (IQR: 47.0–208.8). The monthly family income was less than 7,000 Indian rupees in 27/44 (61.4%) cases, which is less than the cost of second-line ART in a pharmacy in Mumbai (8). Thirty-one (66%) patients were in WHO clinical stage IV at the time of enrolment (9). Three patients were co-infected with HIV and multidrug-resistant TB (MDR-TB). The median duration of exposure to ART of these patients prior to enrolment in this study was 7 years (IQR: 5.0–9.0). Twenty-two (47%) patients had suffered from ARV medication–related grade III or IV toxicity (10).

### Treatment outcomes

**Fig. 1:**
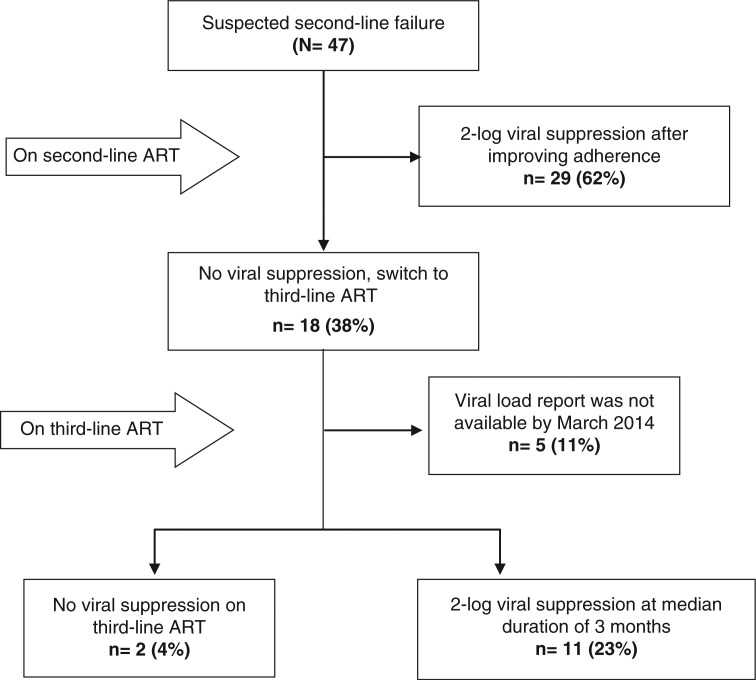
Patients living with HIV and suspected of second-line antiretroviral therapy failure.

Of the 47 patients suspected of second-line ART failure, 29 patients (62%) responded to enhanced adherence support and had a two-log decrease in their level of HIV on subsequent VL testing (Fig. 1). The other 18 patients (38%) did not respond to enhanced adherence support and were eventually switched to third-line ART. The median duration of prior ART exposure of these 18 patients (Table 1) was 9.0 years (IQR: 7.0–10.3). Out of 18 patients who switched to third-line ART, 13 had VL follow-up before the end of March 2014, of whom 11 achieved viral suppression after a median duration of 3 months (IQR: 2.5–3.0). Two patients failed to achieve viral suppression, one of whom died in April 2013 due to *Pneumocystis jirovecii* pneumonia (PCP).

**Table 1:** Clinical characteristics of 18 patients living with HIV and started on third-line ART, Mumbai, India, January 2011–January 2014

	Age	Sex	WHO stage	Prior duration of ART (years)	Comorbidity	Drugs in third-line ART regimen	Duration on third-line ART (months)	Adverse events
1	40	M	III	8	DL, DM	DRV/R, RAL, ATV	30	Transient rise in ALT
2	40	M	IV	8	DL	DRV/R, RAL, EFV, TDF, 3TC	33	No adverse event
3	38	M	IV	8.5	–	DRV/R, RAL, ABC, 3TC	33	No adverse event
4	32	F	III	7	DL	DRV/R, RAL, EFV	32	No adverse event
5	60	F	IV	10	DL, HTN	DRV/R, RAL, ABC	32	No adverse event
6	38	M	IV	7	–	DRV/R, RAL, TDF, 3TC	18	No adverse event
7	40	M	II	10	DL	DRV/R, RAL, TDF, 3TC	30	No adverse event
8	45	M	IV	10	HTN	DRV/R, RAL, TDF, 3TC	15	No adverse event
9	38	F	IV	9	DL	DRV/R, RAL, TDF, 3TC	17	No adverse event
10	49	M	IV	13	DL	DRV/R, RAL, ABC, 3TC	16	No adverse event
11	46	M	IV	4	HBV	DRV/R, RAL, ABC, 3TC	3	No adverse event
12	45	M	IV	16	DL	DRV/R, RAL, ABC, 3TC, EFV	10	No adverse event
13	16	F	IV	6	–	DRV/R, RAL, ABC, 3TC	3	No adverse event
14	40	M	IV	15	DM	DRV/R, RAL, TDF, 3TC	5	No adverse event
15	48	M	IV	9	DM	DRV/R, RAL, ABC, 3TC	6	No adverse event
16	47	M	IV	11	–	DRV/R, RAL, TDF, 3TC	1	No adverse event
17	42	F	IV	6	–	DRV/R, RAL, TDF, 3TC	4	No adverse event
18	52	M	IV	9	–	DRV/R, RAL, TDF, 3TC	3	No adverse event

ART: antiretroviral therapy; DM: diabetes mellitus; DL: dyslipidemia; HTN: hypertension; HBV: hepatitis B; ATV: atazanavir; ABC: abacavir; DRV/R: darunavir boosted with ritonavir; EFV: efavirenz; RAL: raltegravir; TDF: tenofovir; 3TC: lamivudine; ALT: alanine aminotransferase.

## Discussion

As a result of enhanced adherence support and routine HIV VL monitoring, the majority of patients living with HIV who had suspected second-line ART failure in the MSF programme in Mumbai did not have to be switched to third-line ART. In fact, most of the patients suspected to be failing second-line ART in this study had adherence issues rather than HIV strains that were resistant to the ARV drugs, a finding which is in line with the results of previous studies, including a similar South African cohort and several other programmatic cohorts in resource-limited settings (11–13).

One of the main reasons behind the lack of adherence reported by patients was the high cost of second-line ART (prior to enrolment in the MSF programme). These patients had initially opted to receive their HIV treatment in private facilities rather than public ones, in an effort to reduce HIV-related stigma and discrimination; however, they were eventually no longer able to afford the medications and were forced to stop the treatment. Patients co-infected with HIV and TB had a high pill burden, which has the potential to negatively affect adherence. Additional reasons for poor adherence in our cohort included anxiety, in part due to fear of disclosure of their HIV status, and alcohol abuse.

Routine VL monitoring is an important tool to identify poor adherence and ART failure at an early stage, and with proper management this will prevent the accumulation of further resistance mutations and preserve treatment options (14). Routine HIV VL monitoring is not currently available in the public sector in India; instead, CD4 count monitoring continues to be used as the main test to identify treatment failure. VL testing is available within the public sector on only a case-by-case basis, after being approved by an ART centre expert committee. The authors believe that routine HIV VL monitoring should no longer be considered as a luxury, but a necessity in HIV ART programmes, and that it should be rapidly scaled up in the public sector. Efforts should be made to ensure that VL testing is affordable and easily accessible in all resource-limited settings, including the removal of any barriers, such as reliance on a committee to sanction each and every VL test, as this can result in significant delay. Until such time that VL testing is performed routinely in those on ART, actual treatment failure is likely to be grossly underdiagnosed (15).

HIV resistance surveillance or genotyping in patients (16, 17) assists in designing appropriate treatment regimens for patients needing third-line ART. Thus, genotyping should be considered before regimen preparation for all those requiring a switch to a third-line ART regimen. The lack of access to affordable antiretroviral drugs that can be used in robust combinations is another barrier to management of those failing second-line ART. National ART programmes need to be prepared to offer third-line ART (18), and they should already start preparing by making supplies of darunavir and raltegravir accessible. Furthermore, management of patients needing third-line ART can be complicated and may require palliative care (19). A medical dilemma exists for those failing third-line ART regarding whether they should continue costly treatment that consists of many pills and is only weakly effective or switch to another ARV regimen that has a lower pill burden and is less expensive.

The small number of patients in the study is one of the limitations, and therefore generalisation of the results may not be possible. However, this study is one of the few reports from resource-limited settings describing operational feasibility and programmatic challenges in relation to management of patients living with HIV who have suspected second-line ART failure and patients requiring third-line ART. There may be additional factors influencing treatment adherence in such patients; however, it would be difficult to comment on these factors on the basis of routine programme data.

## Conclusion

Early detection of ART failure through routine VL monitoring and HIV resistance surveillance should be prioritised in settings where ART programmes have been successfully established. Adequate adherence support and extensive counselling should be provided to all patients suspected of second-line ART failure since the underlying reason for suspected failure is often not genetic mutation of HIV but improper adherence by patients. HIV viral load testing should be offered after 3 months of enhanced adherence support in all patients with suspected failure based on a single VL measurement.

With intensive psychosocial and medical intervention in those suspected to be failing second-line ART, an unnecessary switch to more expensive and often unavailable third-line ART can be averted in the majority of cases. However, there is still an urgent need for improved access to third-line ART regimens in India, for which the national ART programme should be prepared. The cost of such medications and the additional costs of VL monitoring and genotyping are currently major barriers to the optimal management of patients failing second-line ART.
